# Aortoenteric Fistula: A Differential Diagnosis of Anemia

**DOI:** 10.7759/cureus.42952

**Published:** 2023-08-04

**Authors:** Bruno D Freitas, Antony Dionisio, Beatriz Ferreira, Samuel Azevedo, Inês Araújo

**Affiliations:** 1 Department of Internal Medicine, Hospital de São Francisco Xavier, Lisbon, PRT; 2 Heart Failure Clinic, Department of Internal Medicine, Hospital de São Francisco Xavier, Lisbon, PRT

**Keywords:** abdominal aortic aneurysms, medical management, aortoenteric fistula, anemia, gastrointestinal hemorrhage

## Abstract

Aortoenteric fistulas (AEFs) are a rare and deadly cause of gastrointestinal bleeding that can be easily overlooked, leading to massive bleeding. Secondary AEFs are more common than primary AEFs. An example of a secondary cause of anemia is postoperative hemorrhage due to a surgically placed aortic graft or after endovascular aneurysm repair. This report aims to increase the awareness of AEF as a differential diagnosis when anemia is detected.

The clinical report presents a case of anemia in a 79-year-old man due to a secondary AEF, which occurred in a patient who had undergone abdominal aortic aneurysm surgery 10 years before. Surgical repair is considered the gold standard for AEF treatment; however, in this case, the patient was managed with medical therapy and discharged after two months.

## Introduction

An aortoenteric fistula (AEF) is a rare and life-threatening condition characterized by an abnormal connection between the aorta and the gastrointestinal tract, with an incidence of approximately 0.007 per million people [1]. This connection can cause significant bleeding, which can lead to severe hemorrhage and death. Primary AEFs typically occur as complications of abdominal aortic aneurysms, which may erode into the adjacent intestine and form a fistula. AEFs can also be associated with chronic graft infection or physical stimulation through aortic pulsation pressure contributing to the formation of secondary AEFs [2]. There are two types of secondary AEF, namely, type 1 (most common), termed as true AEF or graft enteric fistula, develops between the proximal aortic suture line and the bowel; type 2, or para-prosthetic enteric fistula, develops without communication between the bowel and the graft. In this type of fistula, bleeding occurs from the edges of the eroded bowel secondary to the mechanical pulsation of the aortic graft [3].

The exact prevalence of AEFs is difficult to determine due to their rarity. However, studies suggest that it is a relatively uncommon condition, accounting for fewer than 1% of cases in patients with aortic aneurysms or aortic grafts [4,5].

Symptoms of AEFs include sudden and severe abdominal pain, gastrointestinal bleeding, and signs of shock [6,7]. Diagnosis is typically made through a combination of clinical evaluation and imaging studies, such as CT scans and angiograms [8]. If left untreated, the mortality approaches 100% [9]. The treatment of AEFs typically involves emergency surgery, aiming to repair the fistula and restore circulation to the affected area. Surgical management often includes excision of the infected or necrotic tissue, repair or replacement of the damaged portion of the aorta, and, if necessary, resection of the affected segment of the intestine [10]. However, the procedure carries a high risk of complications, including infection and bleeding, as well as a high risk of mortality (10-40%), particularly in cases of significant hemorrhage [11].

## Case presentation

The authors present the case of a 79-year-old male patient with pre-existing uncontrolled hypertension, chronic obstructive pulmonary disease (COPD), sarcopenia, poor functional status, suspicion of dementia, and aortic abdominal aneurysm for which he underwent surgery 10 years before (with Dacron bifurcated aortic prosthesis).

The patient presented to the Emergency Department with a two-month history of anorexia and weakness, which worsened in the two days before admission. The patient denied having fever, night sweats, respiratory symptoms, or genitourinary complaints. The physical examination was unremarkable, apart from a slight discomfort on deep abdominal palpation. The blood pressure was 97/52 mmHg and the heart rate was 95 beats per minute. The patient was afebrile and both respiratory rate and peripheral oxygen saturation were within the normal range.

Laboratory evaluation revealed macrocytic anemia with iron, folate, and vitamin B12 deficiencies (Table 1), as well as elevated inflammatory markers.

**Table 1:** Hemogram and iron metabolism evaluation on day one (Emergency Department).

Laboratory parameter	Patient’s result	Normal range
Hemoglobin (g/L)	68	130–170
Mean corpuscular volume (fL)	118	80–96.1
Mean corpuscular hemoglobin (pg)	40.4	27.3–33.7
Serum iron (µg/dL)	32	33–193
Ferritin (ng/mL)	658	30–340
Total iron binding capacity (µg/dL)	217	250–425
Transferrin saturation (%)	15	20–45
Folate (mmol/L)	2.52	10–42
Cyanocobalamin (pmol/L)	80.7	141–489
Prothrombin time (seconds)	15	<14
Activated partial prothrombin time (seconds)	27.8	23–38

**Figure 1:**
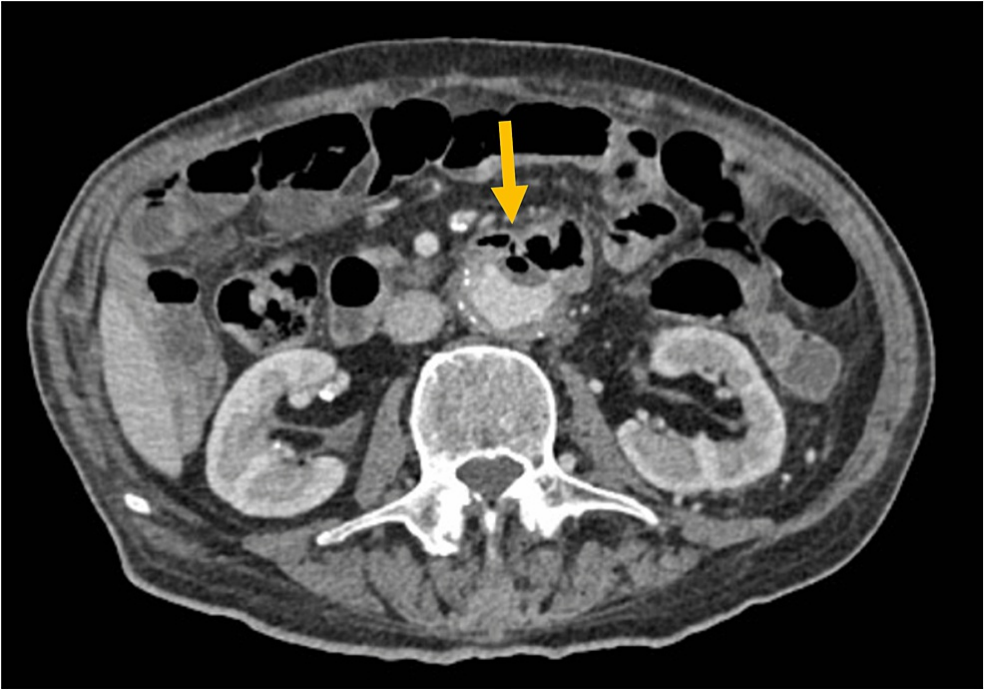
CT scan, infrarenal cut. Arrow pointing to gas in the aortic artery.

**Figure 2:**
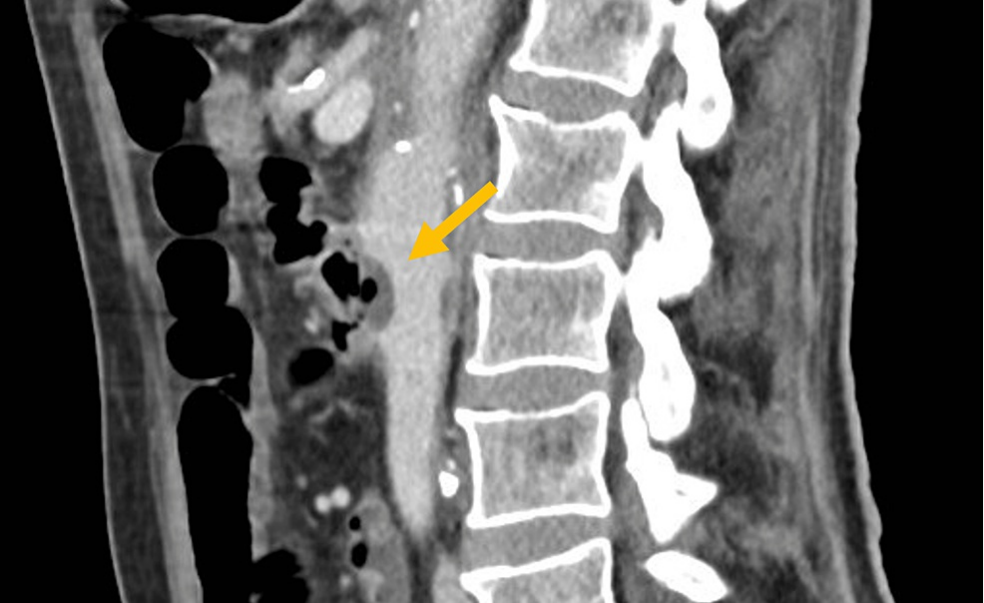
CT scan, sagittal cut (midline). Arrow pointing to gas in the aortic artery.

Due to previous aortic aneurysm surgery, abdominal pain, and de novo anemia, the patient underwent an abdominal CT scan, which revealed an abdominal aorta with aneurysmal dilation and gas bubbles within the aneurysm, inferior to the emergence of renal arteries, in contact with intestinal loop (Figures 1, 2).

After being diagnosed with an AEF, the patient was transferred to another hospital for evaluation by Vascular Surgery. There, both Vascular Surgery and Anesthesiology assessed the patient and deemed him unfit for surgery due to marked sarcopenia, poor functional status, and COPD, being hemodynamically stable and with steady laboratory results. The patient was transferred to the Internal Medicine ward for optimal supportive care, including blood transfusions (total of five packed red blood cells), coagulation optimization, and blood pressure control with calcium channel blocker (amlodipine) to a target of >140/90 mmHg. The patient remained hospitalized for two months where he received a course of antibiotic therapy (piperacillin/tazobactam) with progressive normalization of the inflammatory markers, along with physical rehabilitation. As intercurrence, during the hospital stay, the patient developed diarrhea with isolation of *Clostridium difficile* which was treated with 10 days of oral vancomycin. The patient was later discharged to a nursing home.

## Discussion

This report emphasizes the need for prompt diagnostic testing in patients presenting with acute anemia, as the underlying etiology may have an unfavorable prognosis; however, prompt intervention can lead to a lifesaving outcome. An AEF can be one of these potentially fatal etiologies.

The known pathophysiology of AEF is based on constant irritation from the pulsation of the aortic aneurysm and intestinal luminal stress, leading to ulceration, local necrosis, and, ultimately, fistula formation [12]. There are two types of aortoenteric fistula, namely, primary and secondary. Primary fistulas occur in the absence of a history of previous aortic surgery or trauma, whereas secondary fistulas occur in the context of aortic repair surgery. Bleeding can range from small and self-limited (usually herald bleeding) to hemorrhagic shock. Occasionally, in low-flow fistulas, a thrombus can form and maintain hemodynamic stability [13]. In the present case, a secondary AEF was diagnosed based on a high level of suspicion and confirmed through CT angiography.

Due to the risk of progression to hemorrhagic shock and the high likelihood of surgical intervention, these patients should be closely monitored in a hospital under the care of Vascular Surgery. The treatment of AEF typically involves surgery, followed by intensive care, and, in some cases, antimicrobial therapy with appropriate follow-up management. Due to the significant morbidity associated with open surgical repair, the use of endovascular repair (EVAR) for the treatment of abdominal aortic aneurysms with AEF has increased and can be used as a bridging procedure [14]. In a review analysis, open surgery repair showed an in-hospital mortality rate of 33.9% compared to just 7% with EVAR [15]. Overall, the mortality rate of patients with AEFs is not fully known as survival rates only include patients in whom a diagnosis was made and surgical repair was attempted.

In cases where patients are stable but deemed unfit for surgery due to high intraprocedural mortality, conservative management has been attempted with long-term antibiotics. However, this strategy has also been reported to result in 100% mortality in one year [16].

In this clinical case, as the patient was considered unsuitable for surgery, a conservative approach was adopted. To minimize the risk of bleeding, the patient’s blood pressure was tightly controlled and coagulation was corrected with vitamin K. Additionally, the patient received red blood cell transfusions to maintain hemoglobin levels above 70 g/L. For these reasons, the patient was transferred to the Internal Medicine ward. After the normalization of C-reactive protein and a new study of iron metabolism (Table 2), the patient was treated with ferric carboxymaltose. After spending two months under the care of the Internal Medicine team, the patient was discharged to a nursing home.

**Table 2:** Hemogram and iron metabolism re-evaluation after normalization of inflammatory markers.

Laboratory parameter	Patient’s result	Normal range
Hemoglobin (g/L)	106	130–170
Mean corpuscular volume (fL)	102.2	80–96.1
Mean corpuscular hemoglobin (pg)	34.7	27.3–33.7
Serum iron (µg/dL)	29	33–193
Ferritin (ng/mL)	98	30–340
Total iron binding capacity (µg/dL)	207	250–425
Transferrin saturation (%)	14	20–45
Folate (mmol/L)	>45	10–42
Cyanocobalamin (pmol/L)	748	141–489

## Conclusions

To diagnose an AEF, one should maintain a high index of suspicion in patients with known aortic disease such as aortic aneurysm and especially after open or endovascular surgical repair with unexplained anemia. After the diagnosis, a multidisciplinary approach involving Vascular Surgery, Anesthesiology, Intensive Care, Interventional Radiology, and Internal Medicine should be employed to determine the most suitable treatment plan.

## References

[1] Briggs B, Manthey D (2021). Under the radar: a case report of a missed aortoenteric fistula. Clin Pract Cases Emerg Med.

[2] Mauriac P, Francois MO, Marichez A (2021). Adjuncts to the management of graft aorto-enteric erosion and fistula with in situ reconstruction. Eur J Vasc Endovasc Surg.

[3] Mohammadzade MA, Akbar MH (2007). Secondary aortoenteric fistula. MedGenMed.

[4] Marine L, Mertens R, Torrealba I, Valdés F, Bergoeing M, Vargas F, Yáñez H (2021). [Rupture of abdominal aortic aneurysm into the duodenum: uncommon cause of massive gastrointestinal bleeding]. Rev Med Chil.

[5] Batson RC, Sottiurai VS, Craighead CC (1984). Linton patch angioplasty. An adjunct to distal bypass with polytetrafluoroethylene grafts. Ann Surg.

[6] Smeds MR, Duncan AA, Harlander-Locke MP, Lawrence PF, Lyden S, Fatima J, Eskandari MK (2016). Treatment and outcomes of aortic endograft infection. J Vasc Surg.

[7] Luo J, Tang W, Wang M, Xiao Y, Tan M, Jiang C (2021). Case series of aortoenteric fistulas: a rare cause of gastrointestinal bleeding. BMC Gastroenterol.

[8] Dorosh J, Lin JC (2023). Aortoenterofistula. Radiographics.

[9] Song Y, Liu Q, Shen H, Jia X, Zhang H, Qiao L (2008). Diagnosis and management of primary aortoenteric fistulas--experience learned from eighteen patients. Surgery.

[10] Sanchez JE, Conkling N, Labropoulos N (2011). Compression syndromes of the popliteal neurovascular bundle due to Baker cyst. J Vasc Surg.

[11] Bartley A, Scali ST, Patterson S (2022). Improved perioperative mortality after secondary aortoenteric fistula repair and lessons learned from a 20-year experience. J Vasc Surg.

[12] Champion MC, Sullivan SN, Coles JC, Goldbach M, Watson WC (1982). Aortoenteric fistula. Incidence, presentation recognition, and management. Ann Surg.

[13] Tang SJ, Patnana S, Wu R (2014). Aortoenteric fistula. Video J Encycl GI Endosc.

[14] Rüütmann AM, Kals J (2023). Primary and secondary aortoenteric fistulas in a patient with abdominal aortic aneurysm. Int J Surg Case Rep.

[15] Kakkos SK, Bicknell CD, Tsolakis IA, Bergqvist D (2016). Editor's choice - management of secondary aorto-enteric and other abdominal arterio-enteric fistulas: a review and pooled data analysis. Eur J Vasc Endovasc Surg.

[16] Gunawardena T, Saseekaran B, Abeywickrama S, Cassim R, Wijeyaratne M (2021). Aortoenteric fistula after endovascular aneurysm repair. Case Rep Vasc Med.

